# Multiparametric Magnetic Resonance Imaging Grades the Aggressiveness of Prostate Cancer

**DOI:** 10.3390/cancers14071828

**Published:** 2022-04-05

**Authors:** Juan Morote, Angel Borque-Fernando, Marina Triquell, Anna Celma, Lucas Regis, Richard Mast, Inés M. de Torres, María E. Semidey, Anna Santamaría, Jacques Planas, Luis M. Esteban, Enrique Trilla

**Affiliations:** 1 Department of Urology, Vall d’Hebron Hospital, 08035 Barcelona, Spain; mtriquell@vhebron.net (M.T.); acelma@vhebron.net (A.C.); lregis@vhebron.net (L.R.); jplanas@vhebron.net (J.P.); etrilla@vhebron.net (E.T.); 2 Department of Surgery, Universitat Autònoma de Barcelona, 08193 Barcelona, Spain; 3 Department of Urology, Hospital Universitario Miguel Servet, IIS-Aragon, 50009 Zaragoza, Spain; aborque@comz.org; 4 Department of Radiology, Vall d’Hebron Hospital, 08035 Barcelona, Spain; rmast@vhebron.net; 5 Department of Pathology, Vall d’Hebron Hospital, 08035 Barcelona, Spain; itorres@vhebron.net (I.M.d.T.); mesemidey@vhebron.net (M.E.S.); 6 Department of Morphological Sciences, Universitat Autònoma de Barcelona, 08193 Barcelona, Spain; 7 Vall d’Hebron Research Institute, 08035 Barcelona, Spain; anna.santamaria@vhir.org; 8 Department of Applied Mathematics, Escuela Universitaria Politécnica La Almunia, Universidad de Zaragoza, 50100 Zaragoza, Spain; lmeste@unizar.es

**Keywords:** prostate cancer, aggressiveness, magnetic resonance imaging, PI-RADS

## Abstract

**Simple Summary:**

Prostate cancer (PCa) aggressiveness can be assessed from clinical and pathologic surrogate endpoints as the International Society of Uropathology grade group (GG) in prostate biopsies, the type of pathology from surgical specimens, the clinical stage, and the risk of biochemical recurrence after treatment of localised PCa. Low evidence exists about the association between prostate magnetic resonance imaging (MRI) and PCa aggressiveness beyond the known increased risk of clinically significant PCa (csPCa) as Prostate Imaging and Data Report System (PI-RADS) increase. Therefore, we confirm previous data and generate new evidence from all the previous surrogate endpoints of PCa aggressiveness, confirming that MRI grades the aggressiveness of PCa.

**Abstract:**

We sought to find further evidence showing the increase in PCa aggressiveness as PI-RADS score increases from four surrogates of PCa aggressiveness: i. prostate biopsy GG (≤3 vs. >3), ii. type of pathology in surgical specimens (favourable vs. unfavourable), iii. clinical stage (localised vs. advanced), and risk of recurrence of localised PCa after primary treatment (low-intermediate vs. high). A group of 692 PCa patients were diagnosed after 3-T multiparametric MRI (mpMRI) and guided and/or systematic biopsies, showing csPCa (GG ≥ 2) in 547 patients (79%) and insignificant PCa (iPCa) in 145 (21%). The csPCa rate increased from 32.4% in PI-RADS < 3 to 95.5% in PI-RADS 5 (*p* < 0.001). GG ≥ 3 was observed in 7.6% of PCa with PI-RADS < 3 and 32.6% in those with PI-RADS > 3 (*p* < 0.001). Unfavourable pathology was observed in 38.9% of PCa with PI-RAD < 3 and 68.3% in those with PI-RADS > 3 (*p* = 0.030). Advanced disease was not observed in PCa with PI-RADS ≤ 3, while it existed in 12.7% of those with PI-RADS > 3 (*p* < 0.001). High-risk recurrence localised PCa was observed in 9.5% of PCa with PI-RADS < 3 and 35% in those with PI-RADS > 3 (*p* = 0.001). The PI-RADS score was an independent predictor of all surrogates of PCa aggressiveness as PSA density. We confirmed that mpMRI grades PCa aggressiveness.

## 1. Introduction

Early detection of clinically significant prostate cancer (csPCa) reduces the mortality of PCa [1]. The classic diagnostic approach for PCa, based on systematic prostate biopsies after suspected PCa, has been criticised due to the high rates of unnecessary prostate biopsies and over-detection of insignificant PCa (iPCa) [2]. Recent improvement in early detection of csPCa has come from multiparametric magnetic resonance imaging (mpMRI) and guided biopsies of suspicious detected lesions [3]. This new diagnostic approach takes advantage of the high negative predictive value of mpMRI [4] and the increased csPCa sensitivity of guided biopsies [5]. The current decision-making with prostate biopsies is based on the Prostate Imaging-Report and Data System (PI-RADS) [6], and clinicians usually avoid prostate biopsies when the PI-RADS score is <3 due to the high negative predictive value of mpMRI for csPCa, reaching up to 95% [4]. In contrast, prostate biopsies are always recommended in men with PI-RADS > 3, due to the risk of csPCa higher than 50% in men with PI-RADS 4 and around 90% in those with PI-RADS 5 [7]. Finally, PI-RADS 3 is an uncertain scenario in which the likelihood of csPCa does not reach 20% and the over-detection of iPCa exceeds 50% of tumours detected [7]. Prostate-specific antigen (PSA) density, predictive models, and modern markers are recommended for the proper selection of candidates for prostate biopsy [8,9].

Each increase in the PI-RADS score is usually associated with higher grade group (GG) tumours in the prostate biopsies [7,10], and more advanced tumours have been observed in surgical specimens [11,12]. Based on this evidence, usually it is accepted that csPCa aggressiveness increases across PI-RADS categories; however, there is lack of evidence about other surrogates of PCa aggressiveness. We hypothesize an increase in clinical stage risk of recurrence of localised tumour after primary treatment as PI-RADS increases [3]. Higher evidence generation will assure clinician decisions in the important areas of early detection of csPCa and active surveillance. As an example, failing to detect 10% of csPCa in men with PI-RADS 3 may be reasonable, but not in PI-RADS 5. This suggestion would be based on the greater aggressiveness of tumours detected in PI-RADS 5 beyond the already known greater absolute number of csPCa.

Our aim was to demonstrate the increase in PCa aggressiveness across the PI-RADS score in all known surrogate endpoints: i. the GG pattern in prostate biopsies, ii. the type of pathology in surgical specimens, iii. the risk of recurrence of clinically localised PCa after primary treatment, and iv. the clinical stage of PCa detected.

## 2. Materials and Methods

### 2.1. Design, Setting, and Participants

This was a retrospective analysis of 692 PCa tumours detected in 1486 consecutive men with suspected PCa, serum PSA of ≥3.0 ng/mL and/or abnormal digital rectal examination (DRE), in whom mpMRI was performed before guided and/or systematic prostate biopsies carried out in one academic institution between 1 January 2016 and 31 December 2019. Data were prospectively collected in the early PCa detection program database according to the Standard of Reporting for MRI-Targeted Biopsy Studies (START) [13]. Men taking 5-alpha reductase inhibitors for symptomatic benign prostatic hyperplasia, men with previous PCa detection, and those with incomplete datasets were excluded. Written consent was granted by all participants, and the institutional review board approved the project (PR-AG-02/2021).

### 2.2. MpMRI Technique and Evaluation

MRI scans were acquired with a 3-Tesla scanner with a standard surface phased-array coil, Magnetom Trio (Siemens Corp., Erlangen, Germany). The acquisition protocol included T2-weighted imaging (T2W), diffusion-weighted imaging (DWI), and dynamic contrast-enhanced (DCE) imaging, according to European Society of Urogenital Radiology guidelines [14]. Two expert radiologists reported mpMRI results according to PI-RADS v. 2.0 [10].

### 2.3. Prostate Biopsy Procedure

All men underwent 2- to 4-core transrectal ultrasound (TRUS) fusion-cognitive guided biopsies of PI-RADS > 3 lesions as well as 12-core TRUS systematic biopsies, and only 12-core TRUS systematic biopsies were performed in men with PI-RADS < 3. All biopsies were performed by one experienced urologist using a BK Focus 400 ultrasound scanner (BK Medical Inc., Herlev, Denmark).

### 2.4. Pathologic Analysis and csPCa Definition

Biopsy samples were sent separately to the pathology department where two expert pathologists analysed them, assigning the International Society of Uro-Pathology (ISUP) GG when PCa was detected. csPCa was defined when GG ≥ 2 [15,16].

### 2.5. Measurement of PCa Aggressiveness

The aggressiveness of PCa was assessed from the four available surrogate endpoints: GG pattern in prostate biopsies, type of pathology in surgical specimens, risk of recurrence after primary treatment of localised PCa, and clinical stage.

Relative to GG in prostate biopsies, the distribution of GG, the mean GG estimation, and the rate of GG > 3 were analysed [16,17]. The pathology in surgical specimens was defined as favourable when GG ≤ 2 and pT < 3, and unfavourable pathology when GG > 2 or pT ≥ 3 [18]. The TNM classification of malignant tumours from the International Union Against Cancer (UICC, 8th edition, 2017) was used for clinical staging. The cT is based on DRE information; the cN and cM are based on abdominopelvic and thoracic computed tomography and 99-technetium bone scintigraphy, which are recommended when serum PSA is greater than 10 ng/mL or GG in prostate biopsy is >2. The clinical stage was recodified to localised PCa (T≤3N0M0), locally advanced PCa (T>3N0-1M0), and metastatic PCa (T1-4N0-1M1). Advanced PCa was considered when locally advanced or metastatic disease was detected [3]. The EAU recurrence risk of localised PCa after primary treatment, based on the D’Amico risk established in 1998 [19], was defined as low, intermediate, or high [3]. The four surrogates of PCa aggressiveness were analysed according to the PI-RADS categories ≤ 2, and 3–5, and after grouping PI-RADS ≤ 3 and >3.

### 2.6. Statistical Analysis

Quantitative variables were expressed as medians and 25–75 percentiles, and qualitative variables as rates. Associations between qualitative variables were assessed with the Chi-square test and Fisher correction if needed. The Mann–Whitney U test was used to compare quantitative variables between two groups, and the Kruskal–Wallis test was used when three or more groups were compared. The odds ratios and 95% confidence intervals were obtained. Binary logistic regressions were performed to find independent predictors and receiver operating characteristic (ROC) curves and areas under the curve (AUC) to assess and compare efficacies of predictors. Statistically significant differences were considered when two-side *p* values < 0.05 were found. Analyses were performed with SPSS v.25 (IBM, Statistical Package for Social Sciences, San Francisco, CA, USA).

## 3. Results

The clinical characteristics of 1486 men with suspected PCa due to serum PSA ≥ 3.0 ng/mL and/or abnormal DRE, from whom 692 PCa tumours were detected, are presented in Table 1. We note the overall PCa detection rate was 46.6%, including 36.8% csPCa and 9.8% iPCa. Descriptions and a comparison of characteristics of men with benign tissue in prostate biopsies, iPCa, and csPCa are displayed in Table S1. We note that men with csPCa exhibited higher median age, serum PSA, PSA density, and rate of abnormal DRE than those with iPCa and benign tissue (*p* < 0.001). Conversely, the median prostate volume was lower in men with csPCa (*p* < 0.001). A decreasing trend of repeat biopsies (*p* = 0.910) and an increasing trend of PCa family history (*p* = 0.296) regarding men with benign tissue, iPCa, and csPCa were observed. Finally, different mixes of PI-RADS categories were observed between the three subsets of men. A decreased rate of negative mpMRI (PI-RADS < 3) and increased rate of PI-RADS > 3 were observed in men with csPCa compared to those with iPCa and those with benign tissue (*p* < 0.001).

**Figure 1:**
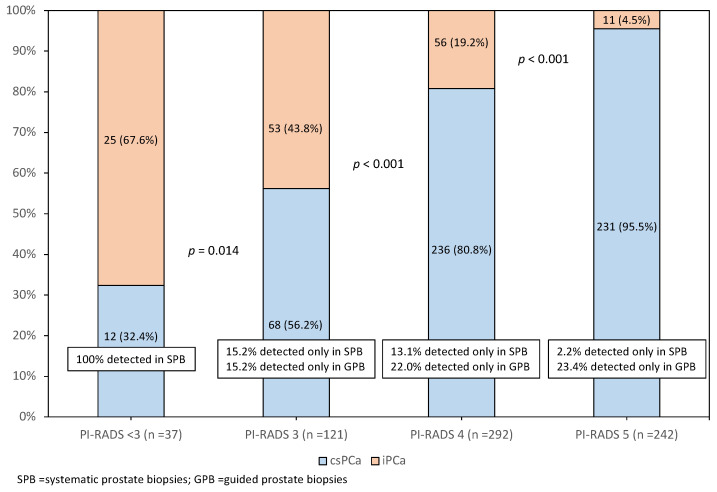
Distribution of csPCa and iPCa according to the PI-RADS categories among the 692 PCa tumours detected and complementarity of systematic and guided biopsies.

The observed csPCa rate increased as PI-RADS category increased (*p* < 0.001), as shown in Figure 1. We observed csPCa in 32.4% of the 37 PCa detected in men with PI-RADS < 3, 56.2% of the 121 PCa detected in men with PI-RADS 3 (*p* = 0.014), 80% of the 292 PCa detected in men with PI-RADS 4 (*p* < 0.001), and 95.5% of the 242 PCa detected in men with PI-RADS 5 (*p* < 0.001). Additionally, we observed an increased rate of csPCa detected exclusively in guided biopsies and systematic biopsies in PCa detected in men with PI-RADS ≥ 3. Complementarity was observed in both types of biopsies, although systematic biopsies detected less csPCa as PI-RADS increased, *p* < 0.001.

GG pattern increased across the PI-RADS categories (*p* < 0.001), as shown in Table 2. The absolute number of higher GG was observed as PI-RADS category increased. The mean GG increased from 1.5 (95% CI: 1.2–1.8) in PI-RADS < 3, to 1.9 (95% CI: 1.7–2.0) in PI-RADS 3 (*p* = 0.017), 2.4 (95% CI: 2.3–2.6) in PI-RADS 4 (*p* <0.001), and 3.5 (95% CI: 3.3–3.6) in PI-RADS 5.

An overall significant association between the distribution of clinically localised, locally advanced, and metastatic PCa and the PI-RADS category was observed (*p* = 0.012), as shown in Table 3. While all tumours detected in men with PI-RADS < 3 were clinically localised, 0.7% of locally advanced PCa and 1.4% of metastatic PCa were detected in the tumours with PI-RADS 4, and 16.1% and 9.5% respectively in tumours with PI-RADS 5 (*p* = 0.012). Significant overall association between the risk of recurrence after primary treatment and PI-RADS score was observed among the 624 cases of clinically localised PCa (*p* < 0.001), as shown in Table 3. We noted that low-risk rate decreased across PI-RADS categories, while high-risk rate increased (*p* < 0.001). The rate of favourable pathology decreased from 100% in tumours with PI-RADS < 3 to 10% of those with PI-RADS 5 among the 234 surgical specimens analysed, while the rate of unfavourable pathology increased from 43.8% in tumours with PI-RADS < 3 to 90% in those with PI-RADS 5 (*p* < 0.001), as shown in Table 3.

To summarise the previous data, Table 4 lists the distribution of PCa tumours according to the four aggressiveness surrogates analysed, by grouping PI-RADS category (≤3 and >3). We note the rate of the GG ≥ 3 pattern increased from 7.6% in PI-RADS < 3 to 32.6% in PI-RADS > 3 (*p* < 0.001), OR 4.9 (95% CI: 3.2–6.9). The rate of high-risk localised tumours increased from 9.5% to 35%, respectively, (*p* = 0.001), OR 3.7 (95% CI: 2.0–6.8). While clinically advanced PCa was not observed in PCa with PI-RADS ≤ 3, up to 12.7% was observed in PCa with PI-RADS > 3 (*p* <0.001), OR 1.1 (95% CI: 1.1–1.2). Finally, unfavourable pathology was observed in 38.9% of surgical specimens of PCa with PI-RADS ≤ 3 and 68.3% of those with PI-RADS > 3 (*p* = 0.030), OR 1.9 (95% CI: 1.1–8.1).

Finally, binary logistic regression analyses to assess independent predictors of PCa aggressiveness among age, PCa family history, type of biopsy (initial vs. repeat), PSA density, and PI-RADS score were conducted. PI-RADS category and PSA density were independent predictors of all PCa aggressiveness surrogates. The behaviour of PI-RADS score and PSA density as predictors of GG pattern ≥ 3 in prostate biopsies (see Table S2), high-risk of clinically localised PCa (see Table S3), clinically advanced PCa (see Table S4), and adverse pathology in surgical specimens (see Table S5) are presented in Supplementary Material. Additionally, the predictive model sharing PI-RADS score and PSA density for GG pattern > 3 was developed (see Table S6), and individualised likelihoods generated showed higher efficacy than PI-RADS and PSA density alone in the ROC curves, as their individual AUC of 0.690 (0.645–0.743) of PSAD, and 0.743 (0.702–0.784) of PI-RADS increased to an AUC of 0.777 (0.740–0.815) when both parameters were joined to predict GG > 3 in PCa. The behaviour of the model sharing PSA density and PI-RADS is presented in Figure S1.

## 4. Discussion

We conducted this study to obtain further evidence that PCa aggressiveness increases with the increased PI-RADS, beyond the known increased probability of csPCa. This is crucial for the early detection of csPCa when prostate biopsy decision-making is based on the PI-RADS category. The current negative predictive value of mpMRI ranges from 80% to 95% [4]. This means that the risk of missing csPCa, if we avoid prostate biopsies in men with PI-RADS < 3, will range between 20% and 5%. However, the aggressiveness of the potentially missed csPCa is also relevant to prostate biopsy decision-making. To fail to diagnose GG 2 PCa is different from missing GG 5 PCa. Similar consideration must be undertaken for prostate biopsy decision-making in men with PI-RADS 4 and 5, in whom the risk of csPCa ranges from 50% to 90% [7]. In addition, the knowledge of tumour aggressiveness associated with PI-RADS category may be helpful in the management of men under active surveillance, because follow-up prostate biopsies could be avoided [20].

This study reports further evidence supporting that high PI-RADS categories (>3) are associated with more aggressive csPCa than low PI-RADS categories (≤3). The current evidence comes from studies on the GG pattern compared with the PI-RADS score in prostate biopsies [7,10]. We have confirmed that the csPCa rate increased as PI-RADS increased, and an increase in higher GG was also observed. Additionally, we observed that the mean GG increased significantly with every PI-RADS category, as well as the rate of tumours with GG > 3 patterns. As in other studies [11,12,21], we have observed more aggressive tumours in surgical specimens from patients with higher PI-RADS categories. We reported results based on the type of pathology assessed from the combination of the highest whole gland GG pattern and the pathologic T stage in around one third of analysed PCa men in whom radical prostatectomy was performed. Unfavourable pathology (GG > 2 and pT > 2) was observed in 68% of tumours detected with PI-RADS > 3, but only in 39% of those detected with PI-RADS ≤ 3.

The clinical stage as a surrogate for PCa aggressiveness according to the PI-RADS score has never been analysed. We observed up to 12.7% locally advanced or metastatic tumours among those with PI-RADS > 3, while all PCa tumours detected in men with PI-RADS < 3 were localised. In addition, we reported an odds ratio of 3.6 for high risk of recurrence after treating localised PCa in men with PI-RADS > 3 over those with PI-RADS ≤ 3.

Our study reported consistent data to verify that PCa aggressiveness increases as PI-RADS increases [7,10,11,12,22,23]. We confirmed our initial hypothesis, as all four surrogates for PCa aggressiveness analysed exhibited similar behaviour. Additionally, we conducted binary logistic regression analysis for each surrogate of PCa aggressiveness, including various candidate predictors, and confirmed that PI-RADS score and PSA density were independent predictors of tumour aggressiveness. Rahota et al. recently reported that PSA density was a predictor of the aggressiveness of PCa tumours detected in PI-RADS 3 [23].

Our study was sizeable. However, a limitation was the low incidence of csPCa in low PI-RADS categories. The aggressiveness of tumours can be assessed only from their outcomes after treatment; then, the assessment of aggressiveness from surrogate endpoints may not be accurate. However, due to the insufficient follow-up of men with PCa after the introduction of PI-RADS, and especially its last versions, analysing surrogates of aggressiveness is the only way to assess this aim. Unfortunately, the specificity of mpMRI for aggressive tumours is not 100% even in PI-RADS category 5, in which csPCa is present around in 90% of cases [7], but benign pathology as granulomatous prostatitis can have an mpMRI signature similar to that of csPCa [24]. On the other hand, it has been shown how mpMRI is able to maintain a good correlation with the pathological findings in whole gland specimens compared to that observed in prostate biopsies [25]

## 5. Conclusions

The aggressiveness of PCa increases with the PI-RADS score. This information is useful in determining whether prostate biopsies should be performed according to the PI-RADS category, whether tools should be used to improve the selection of prostate biopsy candidates, and what thresholds should be used to ensure adequate sensitivity based on the aggressiveness of the potentially detectable tumours.

## Figures and Tables

**Table 1:** Characteristics of the study population of men with suspected PCa.

Characteristic	Value
Number of cases	1486
Median age, years (IQR)	69 (62–74)
Median total PSA, ng/mL (IQR)	6.0 (4.3–9.2)
Abnormal DRE, *n* (%)	109 (19.2)
Median prostate volume, mL (IQR)	55 (40–76)
Median PSA density, ng/mL/cc (IQR)	0.11 (0.07–0.18)
Repeat biopsy, *n* (%)	133 (23.5)
Family history of PCa, *n* (%)	48 (8.6%)
PI-RADS, *n* (%)	
1–2	315 (21.2)
3	444 (29.9)
4	450 (30.3)
5	277 (18.6)
Overall PCa detection, *n* (%)	692 (46.6)
csPCa detection, *n* (%)	547 (36.8)
iPCa detection, *n* (%)	145 (9.8)

IQR = interquartile range; PI-RADS = prostate imaging report and data system; csPCa = clinically significant prostate cancer; iPCa = insignificant PCa.

**Table 2:** Distribution of the absolute and relative grade groups and the mean grade group according to the PI-RADS categories in 692 PCa tumours.

PI-RADS	Grade Group						*p* Value	Mean GG (95% CI)	*p* Value
	1	2	3	4	5	All			
1–2, *n* (%)	25 (67.6)	8 (21.6)	1 (2.7)	3 (1.7)	0 (0)	37 (5.3)	-	1.5 (1.2–1.8)	-
3, *n* (%)	53 (43.8)	41 (33.9)	18 (14.9)	8 (7.0)	1 (0.8)	121 (17.5)	<0.001	1.9 (1.7–2.0)	=0.017
4, *n* (%)	56 (19.2)	118 (40.4)	69 (23.6)	36 (12.3)	13 (4.5)	292 (42.2)	<0.001	2.4 (2.3–2.6)	<0.001
5, *n* (%)	11 (4.5)	45 (18.6)	61 (25.2)	67 (27.7)	58 (24.0)	242 (35.0)	<0.001	3.5 (3.3–3.6)	<0.001
All	145 (21.0)	212 (30.6)	149 (21.5)	114 (16.5)	72 (10.4)	692 (100)	-	2.5 (2.5–2.6)	-

PI-RADS = prostate imaging report and data system; CI = confidence interval.

**Table 3:** Aggressiveness of PCa based on the clinical stage, the EAU recurrence risk of localised PCa, and the type of pathology in surgical specimens, according to the PI-RADS category.

Aggressiveness of PCa, *n* (%)	PI-RADS				All
	1–2	3	4	5	
Clinical stage (*n* = 692), *p* = 0.012					
Localised	37 (100)	121 (100)	286 (97.9)	180 (74.4)	624 (90.2)
Locally advanced	0 (0)	0 (0)	2 (0.7)	39 (16.1)	41 (5.9)
Metastatic	0 (0)	0 (0)	4 (1.4)	23 (9.5)	27 (3.9)
EAU risk of localised tumours (*n* = 624), *p* < 0.001					
Low	29 (80.0)	61 (50.4)	79 (27.6)	11 (6.1)	180 (28.9)
Intermediate	6 (13.3)	47 (38.8)	115 (40.2)	98 (54.4)	266 (42.6)
High	2 (5.4)	13 (10.7)	92 (32.2)	71 (39.5)	178 (28.5)
Type of pathology in surgical specimens (*n* = 234), *p* < 0.001					
Favourable	6 (100)	27 (53.6)	54 (36.0)	3 (10.0)	90 (38.5)
Unfavourable	0 (0)	21 (43.8)	96 (64.0)	27 (90.0)	144 (61.5)

PCa = prostate cancer; n = number; PI-RADS = prostate imaging-report and data system; EAU = European association of urology; Unfavourable pathology = grade group > 2 and/or pT ≥ 3.

**Table 4:** Aggressiveness of PCa based on the surrogates of dichotomic stratification of the grade group in prostate biopsy, the EAU recurrence risk of localised PCa, the clinical stage, and the type of pathology in surgical specimens, according to the PI-RADS categories 1–3 and 4–5.

Criteria of Aggressiveness	PI-RADS		Odds Ratio (95% CI)	*p* Value
	1–3	4–5		
Grade group > 3, n (%)	12/158 (7.6)	174/534 (32.6)	4.881 (3.177–6.885)	<0.001
EAU high-risk localised PCa	15/158 (9.5)	163/466 (35.0)	3.681 (2.032–6.785)	=0.001
Advanced PCa, n (%)	0/158 (0)	68/534 (12.7)	1.139 (1.086–1.196)	<0.001
Unfavourable pathology	21/54 (38.9)	123/180 (68.3)	1.867 (1.137–8.112)	=0.030

PI-RADS = prostate imaging-report and data system; CI = confidence interval; Advanced PCa = locally advanced and/or metastatic; unfavourable pathology = grade group ≥ 2 and/or pT ≥ 3.

## Data Availability

The data presented in this study are available on request from the corresponding author.

## Supplementary Materials

The following supporting information can be downloaded at: https://www.mdpi.com/article/10.3390/cancers14071828/s1, Figure S1: Receiver operation characteristic curves and areas under the curve of PI-RADS score, PSA density, and the model based on PI-RADS and PSA density, for the prediction of ISUP grade group > 3 in prostate biopsies, Table S1: Binary logistic regression analysis for the prediction of grade group > 3 pattern in prostate biopsies, Table S2: Binary logistic regression analysis for the prediction of high-risk localised PCa, Table S3: Binary logistic regression analysis for the prediction of clinically advanced PCa, Table S4: Binary logistic regression analysis for the prediction of adverse favourable pathology in surgical specimens, Table S5: Binary logistic regression for the prediction of adverse favorable pathology in surgical specimens, Table S6: Binary logistic regression for the generation of predictive model of ISUP grade group > 3, based on PI-RADS score and PSA density.
