# Supplementary material for: Multiparametric Magnetic Resonance Imaging Grades the Aggressiveness of Prostate Cancer
Source: Cancers (Basel). 2022 Apr 5;14(7):1828. doi: 10.3390/cancers14071828 (PMC8997549; doi:10.3390/cancers14071828)
Supplement: Supplementary file 1 [file cancers-14-01828-s001.zip › cancers-1648635-supplementary.pdf]

Table S1. Characteristics of the study population of men with suspected PCa according to the pathology findings of benign tissue, iPCa, and csPCa in prostate biopsies.

Characteristic	Benign tissue	iPCa	csPCa	pValue[1]	pValue[2]
Number of cases	794 (53.4)	145 (9.8)	547 (36.8)	-	-
Median age, years (IQR)	66 (61-72)	69 (63-73)	72 (66-76)	=0.021	<0.001
Median total PSA, ng/mL (IQR)	5.6 (4.2-7.7)	4.8 (3.9-6.8)	7.3 (5.0-12.4)	=0.008	<0.001
Abnormal DRE, n (%)	96 (12.1)	20 (13.8)	213 (38.9)	=0.583	<0.001
Median prostate volume, mL (IQR)	65 (48-86)	52 (40-73)	45 (34-59)	<0.001	<0.001
Median PSA density, ng/mL^2 (IQR)	0.09 (0.07-0.16)	0.09 (0.07-0.17)	0.17 (0.12-0.34)	=0.469	<0.001
Repeat biopsy, n (%)	239 (30.1)	32 (22.1)	117 (21.4)	=0.473	=0.910
Family history of PCa, n (%)	52 (6.5%)	12 (8.3)	63 (11.5)	=0.558	=0.296
PI-RADS, n (%)					
1-2	278 (35.0)	25 (8.3)	12 (2.2)	<0.001	<0.001
3	323 (40.7)	53 (36.6)	68 (12.4)		
4	158 (19.9)	56 (38.6)	236 (43.1)		
5	35 (4.4)	11 (7.6)	231 (42.2)		

iPCa = insignificant prostate cancer; csPCa =clinically significant prostate cancer; IQR =interquartile range; PI-RADS =prostate imaging report and data system; p Value[1] =between benign tissue and iPCa; pValue[2] =between iPCa and csPCa.

Table S2. Binary logistic regression for the prediction of ISUP grade group ≥3 in prostate biopsies.

Predictor	Odds Ratio (95% CI)	p Value
Age, Ref. previous year	1.031 (0.995-1.086)	=0.097
PCa family history, Ref. no	1.094 (0.451-2.650)	=0.843
Type of biopsy, Ref. repeat	0.904 (0.472-1.730)	=0.760
PSA density, Ref. previous 0.01 ng/mL^2	2.693 (1.158-6.263)	=0.021
PI-RADS, Ref. PI-RADS <3	2.568 (1.767-3.733)	<0.001

PCa =prostate cancer; PSA =prostate-specific antigen; PI-RADS =prostate imaging-report data system; CI =confidence interval; Ref. =referenced to.

Table S3. Binary logistic regression for the prediction of high-risk localised PCa.

Predictor	Odds Ratio (95% CI)	p Value
Age, Ref. previous year	1.069 (0.998-1.124)	=0.084
PCa family history, Ref. no	1.766 (0.437-7.142)	=0.425
Type of biopsy, Ref. repeat	1.164 (0.492-3.420)	=0.596
PSA density, Ref. previous ng/mL^2	3.756 (1.731-8.832)	<0.001
PI-RADS, Ref. PI-RADS <3	1.859 (1.067-3.237)	=0.029

PCa =prostate cancer; PSA =prostate-specific antigen; PI-RADS =prostate imaging-report data system; CI =confidence interval; Ref. =referenced to.

Table S4. Binary logistic regression for the prediction of clinically advanced PCa.

Predictor	Odds Ratio (95% CI)	p Value
Age, Ref. previous year	1.056 (0.995-1.120)	=0.073
PCa family history, Ref. no	1.321 (0.895-4.197)	=0.075
Type of biopsy, Ref. repeat	1.121 (0.432-2.321)	=0.697
PSA density, Ref. previous 0.01 ng/mL^2	4.633 (1.828-7.741)	**<0.001**
PI-RADS, Ref. PI-RADS <3	1.784 (1.128-4.244)	**=0.019**

PCa =prostate cancer; PSA =prostate-specific antigen; PI-RADS =prostate imaging-report data system; CI =confidence interval; Ref. =referenced to.

Table S5. Binary logistic regression for the prediction of adverse favorable pathology in surgical specimens.

Predictor	Odds Ratio (95% CI)	p Value
Age, Ref. previous year	1.009 (0.941-1.134)	=0.227
PCa family history, Ref. no	0.63 (0.154-2.864)	=0.582
Type of biopsy, Ref. repeat	1.328 (0.377-4.680)	=0.260
PSA density, Ref. previous ng/mL^2	1.270 (1.017-9.920)	**=0.034**
PI-RADS, Ref. PI-RADS <3	3.841 (1.395-8.318)	**=0.005**

PCa =prostate cancer; PSA =prostate-specific antigen; PI-RADS =prostate imaging-report data system; CI =confidence interval; Ref. =referenced to.

Table S6. Binary logistic regression for the generation of predictive model of ISUP grade group >3, based on PI-RADS score and PSA density.

Predictor	Odds Ratio (95% CI)	p Value
PI-RADS score, Ref. to previous one	3.363 (2.512-4.503)	<0.001
PSA density, Ref. to previous ng/mL^2	1.983 (1.138-3.456)	=0.016

PI-RADS =prostate imaging-report data system; PSAD =prostate-specific antigen density; CI =confidence interval; Ref. =referenced to.

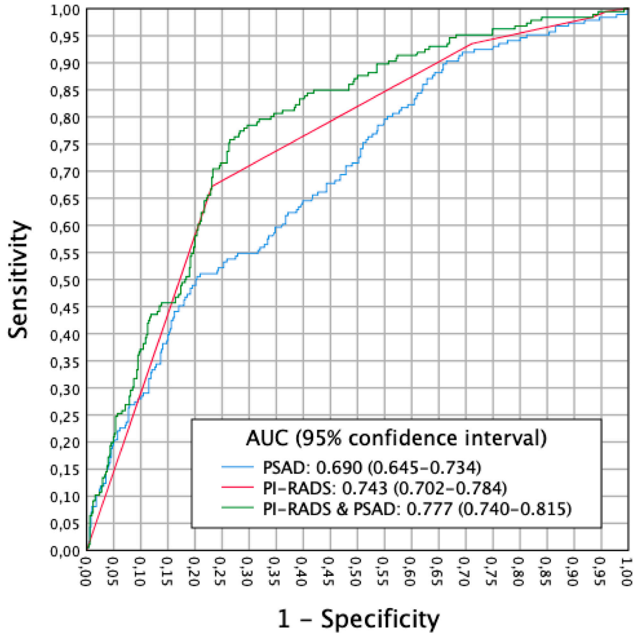

Figure S1. Receiver operation characteristic curves and areas under the curve of PI-RADS score, PSA density, and the model based on PI-RADS and PSA density, for the prediction of ISUP grade group >3 in prostate biopsies.
